# Acidic *Versus* Alkaline Bacterial Degradation of Lignin Through Engineered Strain *E. coli* BL21(Lacc): Exploring the Differences in Chemical Structure, Morphology, and Degradation Products

**DOI:** 10.3389/fbioe.2020.00671

**Published:** 2020-06-30

**Authors:** Gabriel Murillo Morales, Sameh S. Ali, Haibing Si, Weimin Zhang, Rongxian Zhang, Keyvan Hosseini, Jianzhong Sun, Daochen Zhu

**Affiliations:** ^1^Biofuels Institute, School of Environmental Science and Safety Engineering, Jiangsu University, Zhenjiang, China; ^2^State Key Laboratory of Applied Microbiology Southern China, Guangdong Provincial Key Laboratory of Microbial Culture Collection and Application, Guangdong Open Laboratory of Applied Microbiology, Guangdong Institute of Microbiology, Guangdong Academy of Sciences, Guangzhou, China; ^3^Botany Department, Faculty of Science, Tanta University, Tanta, Egypt; ^4^School of Chemistry and Chemical Engineering, Jiangsu University, Zhenjiang, China; ^5^School of Public Affairs, University of Science and Technology of China, Hefei, China

**Keywords:** lignin, *E. coli* BL21(Lacc), biodegradation compounds, acid/alkaline incubation, depolymerization/repolymerization

## Abstract

There is increasing interest in research on lignin biodegradation compounds as potential building blocks in applications related to renewable products. More attention is necessary to evaluate the effects of the initial pH conditions during the bacterial degradation of lignin. In this study we performed experiments on lignin biodegradation under acidic and mild alkaline conditions. For acidic biodegradation, lignin was chemically pretreated with hydrogen peroxide. Alkaline biodegradation was achieved by developing the bacterial growth on Luria and Bertani medium with alkali lignin as the sole carbon source. The mutant strain *Escherichia coli* BL21(Lacc) was used to carry out lignin biodegradation over 10 days of incubation. Results demonstrated that under acidic conditions there was a predominance of aliphatic compounds of the C_3_–C_4_ type. Alkaline biodegradation was produced in the context of oxidative stress, with a greater abundance of aryl compounds. The final pH values of acidic and alkaline biodegradation of lignin were 2.53 and 7.90, respectively. The results of the gas chromatography mass spectrometry analysis detected compounds such as crotonic acid, lactic acid and 3-hydroxybutanoic acid for acidic conditions, with potential applications for adhesives and polymer precursors. Under alkaline conditions, detected compounds included 2-phenylethanol and dehydroabietic acid, with potential applications for perfumery and anti tumor/anti-inflammatory medications. Size-exclusion chromatography analysis showed that the weight-average molecular weight of the alkaline biodegraded lignin increased by 6.75-fold compared to the acidic method, resulting in a repolymerization of its molecular structure. Lignin repolymerization coincided with an increase in the relative abundance of dehydroabietic acid and isovanillyl alcohol, from 2.70 and 3.96% on day zero to 13.43 and 10.26% on 10th day. The results of the Fourier-transformed Infrared spectroscopy detected the presence of C = O bond and OH functional group associated with carboxylic acids in the acidic method. In the alkaline method there was a greater preponderance of signals related to skeletal aromatic structures, the amine functional group and the C – O – bond. Lignin biodegradation products from *E. coli* BL21(Lacc), under different initial pH conditions, demonstrated a promising potential to enlarge the spectrum of renewable products for biorefinery activities.

## Introduction

Due to its abundance and complex polymeric structure, lignin represents a potential biomass resource for use in biorefinery activities (Saito et al., 2013). However, because of its intrinsic recalcitrane to depolymerization, its economical exploitation is still not feasible. Lignin’s primary use is as energy source to produce heat and electricity for the paper and pulp industries (Chen and Wan, 2017; Van den Bosch et al., 2018). The microbiological partial degradation of lignin could represent a promising potential alternative to achieve a feasible production of renewable chemicals (Kosa and Ragauskas, 2013; Kohlstedt et al., 2018). Among the key advantages in the use of bacterial inoculation as approach to partial lignin biodegradation are its easy growth on a liquid medium, easy separation from the culture medium (facilitating recovery of the supernatants for analysis), and a flexible range of incubation temperatures. The whole cell bacterial degradation of lignin represents an opportunity to explore its potential as a source for renewable compounds. Taking advantage of the complex polymeric structure of lignin, could potentially produce a series of compounds of high interest for industries such as biofuels, bioplastics, food additives, pharmaceuticals, and others (Cline and Smith, 2017; Vinardell and Mitjans, 2017).

Laccase is among the most well-known ligninolytic enzymes under study. It is present in higher plants, fungi, and bacteria. Lignin partial biodegradation is achieved through redox reactions, via the oxidation of phenolic and non-phenolic structures and through the cleavage of some of the main lignin inter unit linkages such as β-O-4, β-O-5, β-β, etc. (Chatterjee and Saito, 2015), reducing molecular oxygen to water as a by-product. Researchers have demonstrated laccase’s capacity to modify some functional groups of the lignin polymeric structure (Gianfreda et al., 1999; Hallac and Ragauskas, 2014; Constant et al., 2016; Zhu et al., 2018; Perez et al., 2019). Studies on lignin degradation through bacterial laccase have especially focused their attention on the use of mediators, co-substrates, and optimal reaction temperature (Bourbonnais and Paice, 1990; Bourbonnais et al., 1997; Christopher et al., 2014). Additional studies have demonstrated that many microorganisms can display a series of specialized enzymes based on the availability of specific substrates (Kim et al., 2019). Nevertheless, to the best of our knowledge, studies assessing the partial degradation of lignin by microorganisms (in our particular case bacteria) under different pH conditions still remains obscure. Most studies on lignin biodegradation have paid little attention to the characterization of lignin degradation compounds and lignin chemical structure in the context of the pH incubation conditions.

Publications reporting on different lignin degradation compounds using whole-cell bacterial laccase strains under different pH conditions were recently reviewed. A study of lignin biodegradation using the bacterial strain *Comamonas* sp. B-9, isolated from eroded bamboo slips, performed the experiments on lignin mineral salt medium (KL-MSM) at an initial pH of 7.0, at 30°C, for 7 days. Some of the lignin degradation compounds found were isopropanol, 3-methyl-2-butanol and phenethyl alcohol (Chen et al., 2012). Another study on black liquor from rayon grade pulp paper industry reported its discoloring and detoxification with a potential bacterial consortium composed of *Serratia marcescens, Citrobacter* sp., and *Klebsiella pneumonia*. The experimental conditions involved placing a solution of 10% (v/v) black liquor with 1% carbon and 0.5% nitrogen source in 100 mL volume in flasks of 250 mL for 8 days. After the first 24 h, the initial pH started to drop from 8.0 to approximately 6.5. From there the pH progressively rose to approximately 7.5 on day 8. Some of the lignin degradation compounds detected by gas chromatography/mass spectrometry (GC/MS) were carbamic acid, 1,2-benzenedicarboxylic acid and erythropentanoic acid (Chandra et al., 2011).

A study of directed biodegradation of Kraft lignin, without cosubstrates nor chemical pretreatment, was carried out with the bacterial strain *Cupriavidus* basilensis B-8. The incubation was tested along 7 days at 30°C, but the pH was not reported. The study focused on the measurement and characterization of the production of polyhydroxyalkanoates (PHA’s), omitting the characterization of other lignin degradation compounds. The total concentration of PHA’s was 319.4 mg L^–1^, with (S)-3-hydroxy-butanoic acid, 3-hydroxy-butanoic acid, and 3-hydroxybutyric acid in different percentages (Shi et al., 2017). Further studies reviewed reported the identification of value-added compounds from lignin biodegradation by bacteria (Duan et al., 2016; Suman et al., 2016; Zhu et al., 2017; Sapapporn et al., 2018; Shinoda et al., 2019; Yang et al., 2019). A thorough review of literature on lignin biodegradation identified the lack of research providing a systematic comparison of the lignin degradation in both sides of the pH spectrum using the same bacterial strain. Conducting such a study is necessary to advance research on lignin biodegradation by examining the resulting degradation compounds and the changes to lignin morphology, chemical structure, and molar mass accordingly.

To contribute to this gap in the research, our research group employed a recombined strain of *Escherichia coli* BL21, produced through an intracellular harboring of a laccase gene from the bacterial strain of *Bacillus ligniniphilus* L1, which was previously isolated from the sediments of the South China Sea (Zhu et al., 2014; Zhu et al., 2017). Preliminary results suggest that this mutant bacterial strain has potential to aid in the development of innovative strategies for lignin bacterial degradation. Based on this demonstrated potential, one scientific question must be answered: How do lignin chemical structure, morphology, and molar mass, and lignin degradation compounds produced from the partial degradation activity of the mutant strain *E. coli* BL21(Lacc) differ under acidic and mild alkaline consitions? To answer this question, two different samples of lignin were used: (1) alkali lignin without any chemical pretreatment and (2) alkali lignin which underwent a chemical pretreatment. The initial pH conditions of the samples trigger to drive the pH values of the bacterial growth media to suitable conditions along the incubation. The samples were analyzed for characterization including optical density (OD_280_), measurements of pH, morphology using scanning electron microscopy (SEM), changes in molar mass using size-exclusion chromatography (SEC), presence and identification of lignin degradation compounds using GC/MS and differences in the main functional groups using Fourier-transformed infrared spectroscopy (FTIR). Our results revealed remarkable differences in the lignin morphology, the chemical structure of some functional groups and the generation of biodegradation compounds. We performed experiments without the inoculation of the mutant strain *E. coli* BL21(Lacc) as controls. The results are described and discussed below.

## Materials and Methods

### Materials

Reactants including alkali lignin (CAS number 8068-05-1), 2,2′-azino-bis(3-ethylbenzothiazoline-6-sulfonic acid) (ABTS), isopropyl ß-D-thiogalactopyranoside (IPTG), kanamycin sulfate, 1,4-dioxane, pyridine, N,O-Bis(trimethylsilyl) trifluoro-acetamide with trimethylchlorosilane (BSTFA-TMCS), were purchased from Sigma-Aldrich, in St. Louis. MO, United States. Nitrogen gas (N_2_) was purchased from Shanghai Chemical Industry Park Pu River specialty gases Co., Ltd. The rest of the chemicals described to prepare the different bacterial culture media were purchased from Sinopharm Chemical Reagent Co., Ltd. All the reactants were of analytical grade and used without further purification. The mutant strain *E. coli* BL21(Lacc) with a harbored laccase gene was recombinated at the Biofuels Institute of Jiangsu University.

### Pretreatment of Lignin With Hydrogen Peroxide

A 500 mL solution of de-ionized water with alkali lignin at a concentration of 10 g L^–1^ was prepared. The pH of the black liquor was set at 3.0 ± 0.1 by adding HCl to the aqueous solution. Hydrogen peroxide (30% v/v) at a concentration of 141.12 mM was added to the black liquor. A reactor (Weihai Zhengwei Machinery Equipment Co., Ltd., ZKCF-2L 1, China) submitted the substrates to anoxic conditions with dinitrogen gas at a temperature of 140 ± 5.0°C for 20 min at a pressure of approximately 400 kPa. The black liquor was dried in hot air at a temperature (Jintan Medical Equipment Factory, DHG-9245A, China) between 60–80°C for 4 days. The chemically pretreated lignin was used for bacterial degradation under low pH conditions. For the mild alkaline method, lignin was used after overnight vacuum drying at 60°C in a heat drier (YiHeng Scientific Instrument Co., Ltd., BPZ-6033, China) to remove moisture.

### Preparation of Lignin Culture Medium for Acid and Mild Alkaline Degradation

The initial growth of the mutant strain *E. coli* BL21(Lacc) took place on LB medium for approximately 18–24 h. One milliliter of kanamycin sulfate solution (0.01 g mL^–1^) was added before the inoculation and 100 μL of IPTG solution (0.1 mM) after 12 h of inoculation. For the alkaline biodegradation method, ABTS mediator was added to the LB medium before sterilization. After 24 h of incubation, 10 g L^–1^ of alkali lignin (the lignin medium under alkaline conditions) were added.

For the acid method, the bacterial cells were precipitated from the LB culture medium via centrifugation at 10,000 rpm for 20 min. The pellets were transferred to the lignin medium under sterile conditions. The medium MM63 (used as lignin medium for acidic conditions) was prepared according to a method previously reported (Zhu et al., 2017). The medium MM63 consisted of 100 mM KH_2_PO_4_, 75 mM KOH, 15 Mm (NH_4_)_2_SO_4_, 1 mM MgSO_4_ and 3.9 μM FeSO_4_. Before sterilization, 1 g of lignin was added into 100 mL MM63 medium, along with 1 mM of ABTS as mediator. After sterilization (Sanyo, MLS-3750, Japan), 2.5 g of glucose dissolved in 20 mL of deionized water were added into the culture medium and filtered by 0.2 μm pore size filter for organic solutions. Next, H_2_O_2_ was added with a concentration of 0.50 mM and 1 mL of kanamycin sulfate solution (0.01 g mL^–1^). For the mild alkaline method, lignin was used as a single carbon source without the addition of co-substrates. Before inoculations, the initial pH for LB and MM63 media were set at 7.0 ± 0.10.

After incubation, the bacterial cells were precipitated in a centrifuge (Beckman Advanti J-1, United States) at 12,000 rpm for 20 min. Afterward the supernatants were boiled in a heater at 130°C for 20 min. After cooling down the bacterial cells were precipitated again in the conditions described above. The supernatant was recovered, put into a refrigerator at −20°C for 24 h and submitted to freeze drying (Christ Beta 1–8 LD plus, Germany) for 7 days and kept at cool temperature conditions before analysis. For the preparation of samples for GC/MS analysis, the supernatants were not freeze-dried.

### Optical Density Measures

Aliquots of 0.5 mL were centrifuged at 12,000 rpm for 5 min (Eppendorf, 5804R, Germany), boiled at 100°C for 20 min to inactivate and precipitate the bacterial cells and centrifuged again at the same rotational speed and time conditions. Before optical density readings, the samples were diluted in water at a factor of 1/1,000. The wavelength for optical density measures was set at 280 nm (OD_280_) (Beckman DU 800 spectrophotometers, Beckman Coulter, Inc., United States). Deionized water was set as control. Readings were made in triplicate obtaining standard deviations smaller than or equal to 0.016.

### pH Measures

The initial and final pH readings of the lignin media, and the initial and final pH of the chemically pretreated lignin solution with H_2_O_2_ were conducted with a pH meter (INESA, PHS-25, China) with a precision of 0.01. Intermediate readings were made with litmus paper. For litmus paper readings, a pH band of ±0.50 was added throughout the readings.

### Scanning Electron Microscopy

Dried samples were mounted on small adhesive tapes, coated with a gold–palladium alloy, and examined with SEM using 1 μm as reference scale (SEM, JSM-7800F, Japan).

### Gas Chromatography Mass Spectrometry

The general procedures were prepared according to the method described by Zhu et al. (2017), with some modifications. For samples with bacterial activity at low pH, the pH was adjusted to 2.0 using 5 M of an aqueous solution of HCl. For the samples with bacterial activity above pH 7.0, there was no pH adjustment, to avoid inducing chemical modifications in the original compounds derived from the bacterial activity in the lignin medium and their controls. The samples were thoroughly mixed in twice their initial volume (15 mL) of ethyl acetate. The extraction mixture was collected and adjusted to approximately 1 mL via rotary evaporation at 37°C at vacuum, removing any presence of water with anhydrous Na_2_SO_4_. The samples were stored in small glass bottles. Afterward, the samples were evaporated under a stream of dinitrogen gas. The silylation reaction was developed by adding 100 μL of 1,4-dioxane and 10 μL pyridine, followed by the addition of 50 μL of (BSTFA-TMCS). The organic solution was frequently shaked in a water bath at 80°C for 45 min. Before the final preparation of the samples for GC/MS analysis, the solutions were filtered using filter for organic solutions of 0.22 μm pore size and transferred to clean bottles for sampling with a syringe of 1 mL volume. A volume of 1 μL of silylated mixture was injected into the GC-MS equipment (Agilent Technologies, United States). The analytical column connected to the system was a PE–5MS capillary column (20 m × 0.18 mm internal diameter, 0.18 mm film thickness). Helium was used as a carrier gas with a flow rate of 1 mL min^–1^. The column temperature program was 50°C (5 min); 50–280°C (10°C min^–1^, holding time of 6 min). The transfer line and the ion source temperatures were maintained at 200 and 250°C. A solvent delay of 3.0 min was selected. In the full-scan mode, electron ionization mass spectra in the range of 30–550 (m/z) were recorded at an electron energy of 70 eV.

### Size-Exclusion Chromatography

Molar mass analysis was performed in a Shimadzu Series HPLC system (Shimadzu, Japan), equipped with a column oven unit CTO-20AC, a liquid chromatograph unit LC-20AD, a UV/VIS detector SPD-20A and a degassing unit DGU-20A. A column TSKgel GMPWxl, (Tosoh Corporation) was used for the analysis, based on methacrylate copolymer, with a length of 30 cm and a diameter of 7.80 mm, with an average copolymer particle size of 13 μm. The detection system was operated at a 280 nm wavelength, 50°C temperature and an 0.50 mL min^–1^ eluent flow rate. The sample volume was 200 μL. The retention time was adjusted according to the results obtained by the universal calibration procedure. The software used for the determination of the molar mass was provided by Shimadzu LabSolutions GPC.

The universal calibration was made using five different types of NaPSS standards with different molecular weights – 1,690, 16,000, 33,400, 88,700, and 234,800 g mol^–1^. The results were plotted with a logarithmic function of the molecular weight versus retention time. The solution used as eluent was NaNO_3_ at a concentration of 0.1 M at pH 7.0 ± 0.1. The concentration of the dissolved samples, as well as the standards, was 3 mg mL^–1^ with a minimum liquid volume of 5 mL per sample. Before the analysis, the samples were filtered in a 0.22 μm filter for organic solutions. The calculations for the different types of molecular weights (Mw, Mn) were based on the method previously reported (Andrianova et al., 2018).

### Fourier-Transformed Infrared Spectroscopy

A Nicolet Nexus 470 spectrometer FT-IR with IR spectra between 400 and 4,000 cm^–1^ performed measures at room temperature, applying 10 scans in transmission mode, using KBr pellets of 0.1 mm thickness. The weight of the samples were of approximately 1 mg.

### Statistical Analysis

A statistical correlation between optical density and pH values was evaluated. The analysis was made with IBM SPSS Statistics Software version 22. The correlations were considered significant at the 0.05 level.

## Results

### Acidic and Alkaline Lignin Degradation With *E. coli* BL21(Lacc)

**FIGURE 1:**
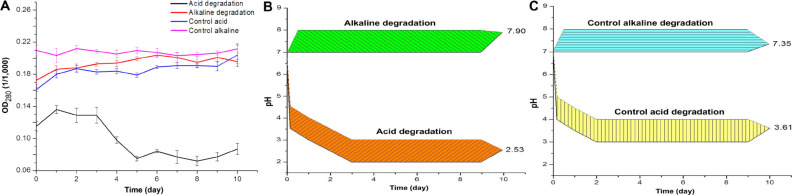
**(A)** Optical density of acid and alkaline-biodegraded lignin and controls at 280 nm. **(B)** pH measures of acid and alkaline-biodegraded lignin and **(C)** pH measures of controls. The range of the pH measures is ±0.50.

The results of optical density and pH measures suggest a modification of lignin chemical structure throughout the incubation time. Figure 1A shows the optical density at the wavelength of 280 nm (OD_280_), which is related to the presence of phenolic groups in lignin (Sharma et al., 2019). For the acidic biodegradation sample, after day 3 the values of OD_280_ dropped, in an interval of 2 days, by 41.8%. In the case of the alkaline biodegraded sample, there was a steady increase in the optical density from day zero to day 6, for a total increase of 18.4%. The results suggest that the main changes on lignin chemical structure occurred after day 5 for the sample under acid biodegradation and after day 6 for the alkaline sample. The optical density values for the control of acidic biodegradation of lignin (hereafter acidic control) were relatively stable, experiencing two increments in the intervals between the days 5 and 6, and days 9 and 10, in 5.29 and 6.86%, respectively. In the control for alkaline biodegradation of lignin (hereafter alkaline control), the variation in the optical density was practically negligible. For the acid control, the initial variations in the optical density, particularly from day zero to day 1, were primarily due to dissolution of lignin in the growth medium. The controls showed no significant changes in optical density.

Figure 1B shows the changes in pH for acidic and alkaline biodegradation of lignin while Figure 1C shows the changes in pH for the controls of acidic and alkaline biodegradation (hereafter acidic and alkaline controls). Represented in Figure 1B, there is a very fast drop in pH in the first hours of reaction for the acidic lignin biodegradation. This might be caused by a chemical equilibrium in the pH between the lignin medium and the chemically pretreated lignin with hydrogen peroxide. From the days zero to 3, a less intense drop in pH occurred. This may be related to the initial bacterial growth and glucose depletion on the lignin medium. After day 3, the values of pH values remained relatively stable. The final pH value for the acidic biodegradation sample was 2.53. In previous literature, few fungal or bacterial strains containing laccase have shown a good capacity for degrading lignin or lignin model compounds under very low pH (Gianfreda et al., 1999; Margot et al., 2013). Our results, however, demonstrated the utility of bacterial laccase for modifying the chemically pretreated lignin under very low pH, comparable with the optimal pH for ABTS activity in the oxidation of lignin model compounds (pH 3.0) (Bourbonnais et al., 1997; Margot et al., 2013).

Figure 1B also shows a single pH band between 7.0 and 8.0 during the 10 days of reaction, suggesting that the ionic equilibrium is more limited in the alkaline range for this particular bacterial strain under the performed experimental conditions. The final pH value was 7.90. This result demonstrates the capacity of the mutant strain *E. coli* BL21(Lacc) to tolerate alkaline conditions to some extent. Our findings of lignin biodegradation in mild alkaline medium are comparable to other reports of lignin degradation and lignin model compounds using bacterial and fungal laccase in the alkaline side of the pH spectrum (Xu, 1997; Ruijssenaars and Hartmans, 2004). Figure 1C shows the pH values for the acidic and alkaline controls. Both results showed similar patterns to those of the pH values of the main samples in Figure 1B. The acidic control also used chemically pretreated lignin and H_2_O_2_ as co-substrate, maintaining a very similar equilibrium in pH values during the first hours of reaction in the lignin medium compared to the of acidic biodegradation sample of lignin along the 10 days of agitation. The final pH value for acidic control was 3.61, 1.08 higher than the value measured for the acidic biodegraded sample, reflecting a difference in the concentration of hydronium ions [(H_3_O^+^)], favorable to the acidic biodegraded sample, of 2.7 mM. The pH values of the alkaline control fluctuated from 7.00 at the beginning of the reaction to 7.35 on day 10. As the pH of alkali lignin in aqueous solution at a concentration of 10 g L^–1^ is higher than 8.0, it is reasonable to assume that the pH could reach an equilibrium value in the first hours of reaction even higher than 7.50, progressively decreasing during the reaction time. A similar reaction is expected in the alkaline biodegraded lignin sample. In conclusion, optical density and pH measurements showed differences between the biodegradation samples and their controls, suggesting a bacterial degradation of lignin during the 10 days of reaction. This conclusion is further confirmed in the analysis below.

### Morphological Description

**FIGURE 2:**
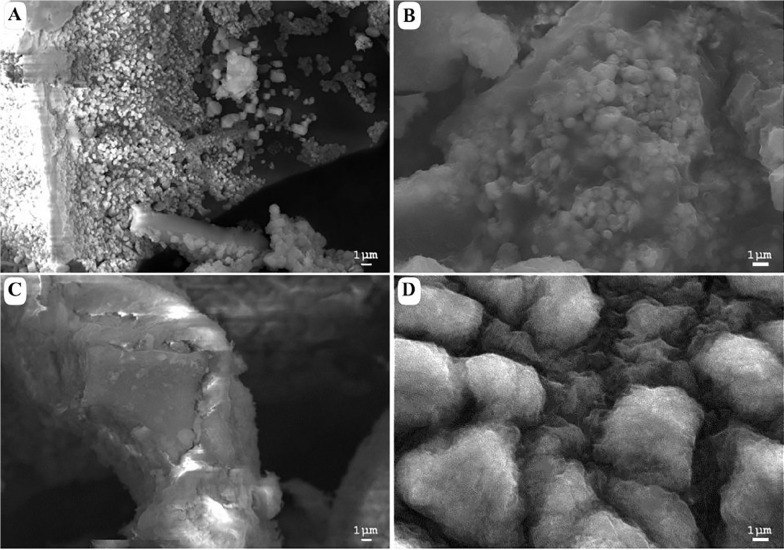
Scanning electron microscopy images of **(A)** acid-biodegraded lignin, **(B)** alkaline-biodegraded lignin, **(C)** control of acid biodegradation, and **(D)** control of alkaline biodegradation. Scale of reference of 1 μm.

Figure 2 shows a morphological comparison using SEM between the samples of acidic and alkaline biodegradation of lignin and their controls, with a scale of reference of 1 μm. Figure 2A shows an acidic biodegradation sample of lignin with blocks in the background coated with a series of crystalline structures of minimum sizes of approximately 1 μm. Figure 2B shows a sample of alkaline biodegradation of lignin with blocks covered by non-crystalline moieties. The average size of the lignin moieties is also of approximately 1 μm. Figure 2C shows a sample of acidic control. Though the acidic control also used chemically pretreated lignin with H_2_O_2_, the scanned image demonstrates the existence of well-defined lignin particles approximately 10 μm in size, without significant damage, and surrounded by foamy-like moieties. In the case of the alkaline control, an absence of clean lignin blocks appears in the background. The lignin moieties have an average size of approximately 5 μm. We conclude that the different pH conditions after lignin modification directly affected the final morphology of the samples. The differences are more notorious for the samples subjected to bacterial activity. Nevertheless, in the case of the alkaline control, there are important modifications on its morphology. In comparison with a typical SEM image of untreated alkali lignin, the acidic control sample suffered less morphological modifications (see Supplementary Figure S1).

### Identification of Lignin Biodegradation Compounds

**FIGURE 3:**
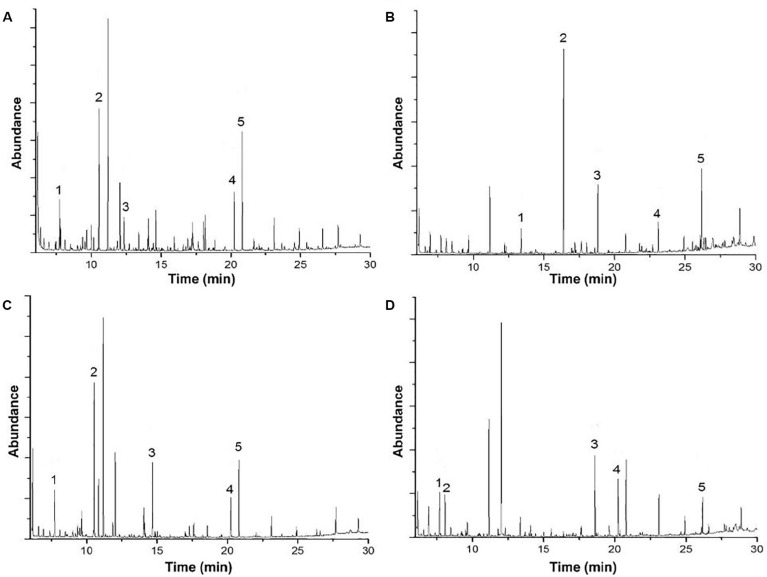
Gas chromatography-mass spectrometry analysis of **(A)** acid biodegradation of lignin, **(B)** alkaline biodegradation of lignin, **(C)** control for acid biodegradation, and **(D)** control for alkaline biodegradation.

Gas chromatography/mass spectrometry analysis gave valuable insights to elucidate if there are differences in lignin degradation compounds under bacterial activity at different pH conditions. A summary of five targeted peaks for each method and their controls, indicating their relative abundance and potential applications, is shown in Table 1. The results of the GC/MS spectral analysis for acidic, alkaline lignin biodegradation, and their controls are shown in Figure 3. The full list of all the compounds – excluding the functional group related to the thioacetolysis reaction – is shown in Supplementary Table S1. It is important to mention that most of the lignin degradation compounds were derived from vanillin, which was identified in a previous GC/MS analysis of alkali lignin – without any chemical pretreatment or bio treatment –, and with a relative abundance of approximately 32% (unpublished).

**TABLE 1:** Summary of main degradation compounds from lignin degradation by *E. coli* BL21(Lacc) and controls.

Acid biodegradation of lignin					Alkaline biodegradation of lignin				
Retention time	Name of compound	Relative abundance (%)	Potential applications	Image of compound	Retention time	Name of compound	Relative abundance	Potential applications	Image of compound
7.73	Crotonic acid	5.22	Precursor for paints and adhesives	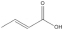	13.38	2-phenylethanol	3.75	Ingredient in flavors and fragances	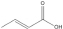
10.53	DL-lactic acid	11.28	Polymer precursor, descaler, anti bacterial agent	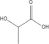	16.38	1H-Indole	32.00	Bacterial signal	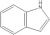
12.33	3-hydroxy butanoic acid	3.32	Copolyester	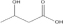	18.80	Isovanillyl alcohol	10.26	Ingredient in flavors	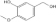
20.23	Vanillic acid	4.83	Flavoring agent	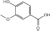	23.10	Hexadecanoic acid	4.33	Substrate for biofuels	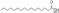
20.82	Protocatechuic acid	9.67	Antioxidant/anti inflammatory	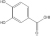	26.17	Dehydroabietic acid	13.43	Anti microbial, anti tumor, anti inflammatory	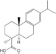
**Control acid biodegradation of lignin**					**Control alkaline biodegradation of lignin**				
7.73	Crotonic acid	6.31	Precursor for paints and adhesives	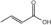	7.73	Crotonic acid	5.46	Precursor for paints and adhesives	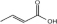
10.55	Propylene glycol	16.48	Thermoplastics, anti freeze, cosmetics	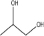	8.09	Ethanamine	5.91	Co-substrate in medical applications	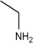
14.68	Succinic acid	7.10	Metabolic intermediate, platform for polymers	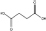	18.60	Apocynin	8.53	Anti inflammatory agent	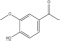
20.23	Vanillic acid	4.34	Flavoring agent	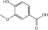	20.23	Vanillic acid	6.11	Flavoring agent	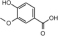
20.82	Protocatechuic acid	7.88	Antioxidant/anti inflammatory	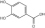	26.17	Dehydroabietic acid	4.47	Anti microbial, anti tumor, anti inflammatory	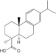

In Table 1 it is the abundant presence of aliphatic compounds with the general structure C_3_–C_4_ namely carboxylic acids in the acidic biodegradation sample, such as crotonic acid (#1), lactic acid (#2), and 3-hydroxybutanoic acid (#3), with relative abundances of 5.22, 11.28, and 3.32%, respectively. The short-chain carboxylic acids have potential applications as substrates for polymers and chemical precursors for paints, adhesives and other compounds (Doi et al., 1988; Rajan et al., 2014; Yang et al., 2018; Hack et al., 2019). Furthermore, two compounds with aromatic structures were also identified: vanillic acid (#4) and protocatechuic acid (#5), with relative abundances of 4.82 and 9.67%. These compounds are associated with the bacterial metabolism of vanillin. Vanillic acid was detected at the retention time of 20.228 min (see Figures 3A,C). Recently, researchers have reported on the use of vanillic acid as building block for copolymers (Gioia et al., 2018). Protocatechuic acid is well-known for its antioxidant capacity (Xi et al., 2016), and thus has potential for use in medicine/pharmaceutics.

The five targeted peaks of the alkaline biodegradation sample are 2-phenylethanol (#1), 1H-indole (#2), isovanillyl alcohol (#3), hexadecanoic acid (#4), and dehydroabietic acid (#5). The relative abundances of each compound were 3.75, 32.00, 10.26, 4.33, and 13.44%, respectively. 2-phenylethanol is used as an ingredient for flavors and perfumery for its rose-like aroma (Sendovski et al., 2010). 1H-indole is a molecule that acts as an important bio signal which is produced from some bacterial strains to regulate the transition from the exponential to the stationary phase. It is also related to a stress response and promotes resistance to a range of antibiotics and toxins through physical export and oxidative-stress protective mechanisms (Lee et al., 2010). Isovanillyl alcohol is an isovanilloid associated with vanillin, which is used as an ingredient in the food industry. Hexadecanoic acid is the only carboxylic acid on the list. It is used as a substrate for biofuels synthesis (Janßen and Steinbüchel, 2014). Dehydroabietic acid is found in resins or extracts of conifers and has being the subject of research for its anti-microbial, anti-tumor and anti-inflammatory properties (González, 2015; Vahermo et al., 2016).

The acidic and alkaline controls showed some similarities and differences compared to the samples under the bacterial activity of *E. coli* BL21(Lacc). The five compounds identified in Table 1 for the acidic control were crotonic acid (#1), propylene glycol (#2), succinic acid (#3), vanillic acid (#4), and protocatechuic acid (#5). The relative abundances represented 6.36, 16.48, 7.10, 4.34, and 7.88%, respectively. Compared with the lignin’s acidic biodegradation sample, it is notable that in the acidic control the compounds in common also have similar relative abundances. For crotonic acid and vanillic acid, this suggests a limited metabolism of those compounds by the mutant bacterial strain. The differences of the lignin degradation compounds between the acidic biodegradation sample and its control are also evident. Some of the most remarkable differences rely on the presence of propylene glycol and succinic acid. On the other hand, the compounds in the acidic biodegradation sample are present in higher amounts, making up between 2.0 and 3.0%. The compounds found only in the acidic biodegradation sample include lactic acid, almost in the same retention time than propylene glycol (10.536 versus 10.55 min), glycerol (14.101 min), and adipic acid (17.235 min), among others (see Supporting Material 2). These results reflect an intense metabolic activity in the chemically modified lignin, which resulted in the degradation of the initially abundant aromatic compounds, suggesting that the addition of glucose as co-substrate, in synergy with the mutant bacterial strain could propitiate a more complex biological effects on lignin.

The alkaline control also exhibited some similarities and differences with the compounds found in the sample of alkaline biodegradation sample. For the alkaline control, are worth mentioning crotonic acid (#1), ethanamine (#2), apocynin (#3), vanillic acid (#4), and dehydroabietic acid (#5). Their relative abundances represented 5.46, 5.91, 8.53, 6.11, and 4.47%. From this list two compounds were also present in the acidic control: crotonic acid and vanillic acid. The differences between the products of the alkaline biodegradation sample were also notable. In the alkaline biodegradation sample, there was a preponderance of three compounds: 1H-indole, isovanillyl alcohol, and dehydroabietic acid. Dehydroabietic acid was also present in the alkaline control, but in the case of the alkaline biodegradation sample, the relative abundance was almost threefold higher. As expected, 1H-indole was not identified in the alkaline control sample, confirming the absence of bacterial activity. 1H-indole is produced as a bio signal for oxidative stress in the bacterial growth medium. Besides the high concentration of 1H-indole, the simultaneous absence of apocynin in the sample under biodegradation confirms the environment of oxidative the bacterial mutant strain was under during lignin oxidation in mild alkaline conditions (Stefanska and Pawliczak, 2008).

Contrasting with the large amount of lignin degradation compounds in the acidic biodegradation sample, the GC/MS analysis detected lower amounts of lignin degradation compounds in the alkaline biodegradation sample (see Figures 3A,B). In Table 1, the differences in their chemical structure are notable. The five targeted peaks of the alkaline biodegradation sample comprised 63.77% of the total detection by GC/MS, nearly double the amount measured in the acid biodegradation sample (32.81%). Most of the relative abundances in the alkaline method came from compounds containing at least one aromatic structure. Some of them originated from lignin degradation by *E. coli* BL 21(Lacc), and one was produced as a bio signal (1H-indole). A carboxylic acid molecule is present at the retention time of 23.107 min (hexadecanoic acid), which was also detected in the acidic method, in both cases a small amount. The presence of this compound in all the samples and their controls (see Supplementary Table S1) requires further study.

Due to their high relative abundances, particular attention was paid to 1H-indole and dehydroabietic acid. Samples were analyzed using GC/MS on the days 0, 3, 7, and 10 to track the evolution of their relative abundances (see Supplementary Table S2). After several hours of the addition of lignin on day zero, the relative abundance of 1H-indole reached 19.71%. It is likely that 1H-indole was already produced before the addition of lignin due to the previous addition of tryptone for the preparation of the LB medium for the initial bacterial growth. On day 7, its abundance peaked at 56.52%, and then decreased to 32.00% on day 10. The initial pH conditions of the alkaline lignin medium could trigger high production of 1H-indole from resistant cells as an assistance to sensitive cells for their survival in the initial incubation conditions. The relative abundance of dehydroabietic acid on day zero was 2.70%, progressively increasing its value during the incubation time, reaching its maximum value of 13.44% on day 10. Interestingly, in the case of isovanillyl alcohol, there was also an increase in its relative abundance, starting with 3.96% on day 0, reaching a maximum value of 12.10% on day 7, and ending on day 10 with 10.26%. All of these compounds have a similar response in their relative abundances throughout the bacterial activity.

In conclusion, most of the lignin degradation compounds produced by the bacterial mutant strain activity of *E. coli* BL21(Lacc) could be conditioned by the evolution of the pH values during lignin biodegradation. The main trend was the production of carboxylic acids at low pH values, and a series of diverse aryl compounds in the alkaline side with an increase for some in their relative abundance under oxidative stress conditions after 7 days of reaction.

### Statistical Analysis

The Shapirov–Wilk test was initially performed to verify whether the optical density and pH values fit a normal probability distribution. The probability values (*p*-values) obtained from the Shapirov–Wilk test in the case of alkaline biodegradation of lignin experiment were greater than 0.05, which implied that the results were normally distributed. The correlation between the optical density and the PH values obtained throughout 11 observations for the experiment for alkaline biodegradation was equal to 0.829, indicating a high significant positive correlation (see Table 2).

**TABLE 2:** Statistical correlation between optical density and pH for acid and alkaline biodegradation of lignin and controls.

Sample	Statistical correlation	Correlation test	Significance level
Acid biodegradation of lignin	0.726	Parametric Pearson	0.01
Control acid	−0.852	Parametric Pearson	0.01
Alkaline biodegradation of lignin	0.829	Non-parametric Spearman	0.01
Control alkaline	−0.227	Parametric Pearson	No significance

The *p*-values obtained from Shapirov–Wilk test for the acidic biodegradation sample of lignin were smaller than 0.05, which indicated that the results did not follow a normal distribution. Thus, we performed the non-parametric Spearman correlation coefficient test for the 11 observations. The test revealed a high significant positive correlation of 0.726 (see Table 2). In the case of the acidic control sample, our results did not follow the normal distribution. Thus, we performed the non-parametric Spearman correlation coefficient test. The test revealed a high significant negative correlation of 0.852. For the results obtained from the of alkaline control sample, there was no significant correlation. The results of this analysis are shown in Table 2.

We conclude that for acidic and mild alkaline degradation of lignin using the mutant strain *E. coli* BL21(Lacc) there was a highly significant positive correlation between the values of OD_280_ and pH throughout the 10 days of reaction.

### Molar Mass Analysis

**FIGURE 4:**
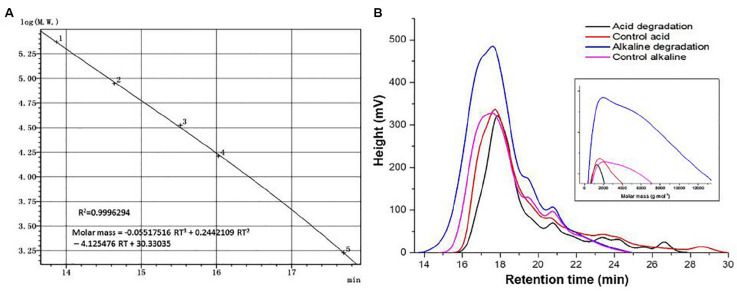
Size exclusion chromatography analysis. **(A)** Universal calibration, **(B)** curves of acid, alkaline biodegradation of lignin, and controls. From the equation of the universal calibration, “RT” means “retention time.”

Molar mass represents an important parameter which reflects quantitative changes on a quantitative basis. The universal calibration curve in Figure 4A shows a very high value in the coefficient of determination, allowing to use its equation for the estimations of M_n_ and M_w_. Figure 4B shows the spectral curves for the samples of acidic and alkaline biodegradation of lignin and their controls, indicating the retention time in the abscissa of the main chart. The augmentated chart of the curves of molar mass shows the equivalent values of the molar mass in the abscissa, in g mol^–1^ (from 0 to 13,000 g mol^–1^). The main chart notes that the curves of the molar mass of acidic biodegradation of lignin and its control have broader late eluting portions (Chatterjee and Saito, 2015), evidence of ionic adsorptions between the lower mola2r mass fractions of lignin and the GPC column particles. In contrast, the of alkaline biodegradation samples of lignin and the alkaline control showed more compact configurations. For the alkaline biodegradation sample, its peak was found in a region of higher molar mass. A similar result was found for the curve of the molar mass of the alkaline control, but more moderate.

Table 3 shows the values of the number-average (M_n_) and weight-average (M_w_) molar mass of the lignin samples. The sample submitted to alkaline biodegradation underwent a 6.75-fold and 3.45-fold increase in its M_w_ and M_n_ values compared to the biodegraded sample in acidic conditions (32,325 and 7,095 g mol^–1^ for alkaline biodegradation of lignin versus 11,254 and 2,054 g mol^–1^ for acidic biodegradation of lignin), and for alkaline biodegradation, the polydispersity index (PDI) was 4.55, a 1.96-fold increase compared to the PDI value of 2.32 for the acidic biodegradation of lignin (Chatterjee and Saito, 2015). It can be inferred that a repolymerization occurred on the molecular structure of the sample of alkaline biodegradation after the incubation time. This might be caused due to the degradation of the lightest molar mass lignin fractions in combination by complex chemical interactions during the bacterial activity of *E. coli* BL21(Lacc), viz., changes in polarity of lignin blocks, rearrangement of non-covalent bonds, etc. Our findings on the increase of lignin’s molar mass are in agreement with previously reported studies (Nugroho Prasetyo et al., 2010; Moya et al., 2011).

**TABLE 3:** Molar mass characterization of lignin samples.

Sample	M_w_	M_n_	PDI
Alkaline biodegradation of lignin	32,325	7,095	4.55
Acid biodegradation of lignin	4,782	2,054	2.32
Control acid	5,996	2,741	2.19
Control alkaline	11,254	4,362	2.58

The acidic control showed slight differences compared to its corresponding biodegraded sample, reaching higher values on its M_n_, and M_w_ values by 25 and 33%, respectively. The PDI value of the acidic biodegraded sample was higher than its control by 5.9%. Interestingly, in the case of the alkaline control there was an increase in the values of M_n_, and M_w_ compared to the acidic control. The alkaline control experienced increments in the M_n_, and M_w_ values by 59 and 87%, respectively. From these results we conclude that a depolymerization occurred in the acidic biodegradation sample of lignin where ring-opening reactions could occur, generating a series of short-chain aliphatic compounds with many carboxylic acids among them. On the other hand, the alkaline conditions could trigger changes in the concentration of the main lignin monomers, which could be attributed to the interactions between reactive functional groups of lignin. This would lead to the rearrangement and reconfiguration of lignin’s overall chemical structure after 10 days of shaking, producing a stronger repolymerization on the lignin degraded sample and a moderate repolymerization on its control.

In order to elucidate lignin repolymerization during the alkaline incubation, we also measured the molar mass on days 1 and 5. Table 4 shows the results. From days 1–5, there was a twofold increase approximately in the values of M_n_ and M_w_ relative to the first day. From days 5 to 10, the increase ratio became larger, in the order by almost fourfold relative to day 5. The PDI values also changed over time, increasing from 2.78 on day 1, to 3.50 on day 5, and to 4.56 on day 10. These results suggest that the alkaline biodegradation of lignin could exert an important influence over the changes in the molar mass of lignin, particularly after the fifth day. Our results suggest that the changes in the molar mass of alkaline biodegradation of lignin could be a consequence of major changes in the lignin’s polymeric structure. The changes in the relative abundance of some compounds reinforces this possibility. The increase of the relative abundance of dehydroabietic acid (molecular weight = 300.4 g mol^–1^) on days 0, 3, 7, and 10, determined by GC/MS analysis, provides a clear example. The abundance of this metabolite was 2.7, 5.23, 13.14, and 13.44%, respectively (see Supplementary Table S2). A similar phenomenon was observed with isovanillyl alcohol.

**TABLE 4:** Change in molar mass for alkaline biodegradation of lignin.

Day	Mn	Mw	PDI
1	2,083	5,798	2.78
5	2,767	9,671	3.50
10	7,095	32,325	4.56

### Structural Characterization

**FIGURE 5:**
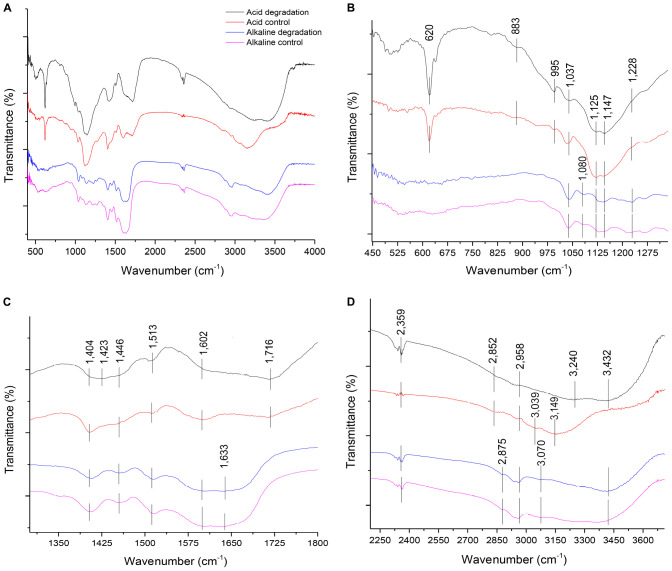
**(A)** Overall FTIR spectra of acid and alkaline biodegradation of lignin and controls; **(B)** spectra from wave numbers 450–1,300 cm^–1^; **(C)** 1,300– 1,800 cm^–1^; **(D)** 2,250–3,700 cm^–1^.

The FTIR analysis method provided insights for a qualitative evaluation of the chemical structure of alkaline and acidic biodegraded lignins and their controls. The analysis confirmed the presence of guaiacyl (G) and syringyl (S) lignins (Gall et al., 2014). Some differences in the presence of functional groups among the lignin preparations were found and the results are shown in Figure 5. Figure 5A displays the overall FTIR spectra, Figure 5B shows more details from the wave numbers 450 to 1,300 cm^–1^, while Figure 5C shows from 1,300 to 1,800 cm^–1^ and Figure 5D shows from 2,250 to 3,700 cm^–1^, respectively.

In Figure 5B, at the wave number of 1,228 cm^–1^, there is a band displayed for the alkaline biodegradation sample and its control, which depicts the stretching of the C – O – C bond, providing important evidence of the stronger presence of aryl ether linkages, viz., β – O – 4′ bond (Yang et al., 2007). In-plane deformations of C – H bonds in G type lignins (in aromatic structures and aliphatic moieties) are evidenced at wave numbers 1,147 and 883 cm^–1^, while the band at 995 cm^–1^ belongs to the deformations out of plane in the – CH = CH – group (Faix, 1991; Sun et al., 2003). The band at 1,080 cm^–1^ is only present in the alkaline samples, corresponding to the stretching of the C – O bond, and reaffirming the previously described at the peak at 1,228 cm^–1^ (Sun et al., 2003). The peak at 620 cm^–1^ possibly describes the presence of a S – O bond, becoming particularly accentuated in the cases of acidic biodegradation of lignin and its control (Nugroho Prasetyo et al., 2010).

The band identified at the wave number 1,716 in Figure 5C is related to the C = O stretch for carboxylic acids. This result fits with the band assigned to the O-H bond for the acidic degradation sample and its control (El Mansouri and Salvadó, 2007; Faix, 1991). The alkaline samples showed a signal at the wave number of 1,633 cm^–1^, assigned to the stretching of the C = C bonds of benzene rings in the IR spectra (Yang et al., 2007). Although is not confirmed, we surmise this could be associated with the repolymerization in alkaline conditions of the lignin biodegraded sample and its control. In contrast, at 1,602 cm^–1^, all the samples displayed aromatic skeletal vibrations in the C – O bonds. This could be related to ether bonds, where the alkaline samples and the acidic control showed higher intensities, evidence of the preservation of ether linkages among aromatic structures (Faix, 1991; Sun et al., 2003; Gillgren et al., 2017). At 1,513 cm^–1^ all the samples had a peak, corresponding to aromatic skeletal vibrations, G > S (Faix, 1991; Gordobil et al., 2016; Yu et al., 2019). The C–H stretch identified at 1,446; 1,423 and 1,404 cm^–1^ are related to the C–H bonds in Figure 5D at the wave numbers 2,958, 2,875, and 2,852 cm^–1^, respectively (Wade, 2013).

As reported in Figure 5D, all the lignin samples showed a broad response in the absorption band related to the O–H bond for aromatic structures at 3,432 cm^–1^ (Cheng et al., 2016), with the exception of the acidic control, which was identified at 3,500 cm^–1^. The acidic biodegradation sample and its control showed a second absorption band between the wave numbers 3,240 and 3,149 cm^–1^. This band is assigned to the stretching of the O–H bond of carboxylic acids. For the biodegradation of lignin under low pH conditions, based on GC/MS data, the relative abundance of the carboxylic acids had an important share in the total detection of all the compounds, confirming the assignment of the wave number to this chemical bond (Yang et al., 2007). The alkaline samples of lignin displayed a signal at 3,070 cm^–1^, which can be attributed to the N–H bond (Wade, 2013). We speculate that the band at 3,039 cm^–1^, particularly strong in the sample of the acid control, could belong to a stronger presence of the stretching of the hybridized sp^2^ C–H bond (Wade, 2013). The signals at the wave numbers 2,958, 2,875 and 2,852 cm^–1^ are related to the asymmetric vibrations and stretching of sp^3^ hybridization of C–H bonds, particularly – CH_3_ and – CH_2_ – groups, associated to aliphatic and aromatic structures (El Mansouri and Salvadó, 2007; Wade, 2013; Yang et al., 2007; Reyes-Rivera and Terrazas, 2017). The band at 2,359 cm^–1^ was assigned to the presence of the nitrile functional group (−C≡N), which was confirmed in the results of GC/MS analysis (see Supplementary Table S1; Wade, 2013).

## Discussion

In this study, alkali lignin was submitted to partial biodegradation by the bacterial mutant strain *E. coli* BL21(Lacc) under acidic and mild alkaline conditions for 10 days. The final outcomes from those treatments were remarkably different in terms of optical density response, pH evolution, morphology, detection of lignin degradation compounds, presence of functional groups, and changes in the molar mass.

The signals detected by the optical density measures at 280 nm, which indicates the presence of aromatic structures in lignin, showed a decay of 41.80% for the acidic lignin biodegradation sample and a peak of 18.40% in the alkaline lignin biodegradation sample. This is evidence that the lignin samples experienced two different chemical structure modification processes. These results indirectly reflected the phenomena of partial depolymerization for the acidic biodegradation and the repolymerization for the alkaline biodegradation of lignin. The existence of two possible modification mechanisms altering the chemical structure of lignin are strongly suggested by the chemical structures present for each case in the lignin biodegradation compounds identified by the GC/MS analysis. In spite of the two different responses in the optical density, in both cases the statistical analysis showed a significant correlation between the OD_280_ and the pH. Furthermore, based on the SEM images, referenced at the scale of 1 μm, the differences in the morphology of the lignin samples were notable. For the acidic biodegradation sample, the lignin blocks were coated on their surfaces by crystalline particles of sizes smaller than 1 μm. The specific lignin degradation compounds associated with that formation merit further studies.

Based on the results of GC/MS analysis, for acidic biodegradation of lignin, a plethora of compounds with aliphatic chemical structures were found, possibly as a result of intense aromatic ring-opening reactions. The majority of these compounds are alkyl compounds of the type C_3_–C_4_, associated to guaiacyl lignin degradation. This was confirmed by the identification of protocatechuic acid as one of the lignin metabolites (Xu et al., 2019). These compounds have potential applications as renewable substrates for food flavorings, biofuels and adhesives, and copolymers for bioplastics, among others. In contrast, most of the lignin degradation compounds identified in the alkaline biodegradation sample showed a tendency to preserve their aromatic structures. Some of these lignin degradation compounds have potential applications as food flavorings and as medical treatments for inflammations, and tumors.

The high concentration of 1H-indole provided clear evidence that the mild alkaline conditions for lignin biodegradation as a single carbon source represented a stressful environment for the activity of the mutant strain *E. coli* BL21(Lacc). In connection with this, a phenomenon of increase in the relative abundance of dehydroabietic acid and isovanillyl alcohol was notorious, which ceased until the production of 1H-indole peaked at the seventh day, suggesting the beginning of the stationary phase in the alkaline lignin incubation.

In the case of acidic biodegradation of lignin, reactions similar to aldol condensation may have occurred. For the alkaline biodegradation of lignin, presumably different condensation reactions were more predominant, especially those dependent on relative higher pH conditions which could activate phenyl groups. It is likely that phenyl intermediates have produced coupling reactions in a small extent of degradation of the aromatic structures, thus, triggering a repolymerization of the lignin backbone, which increased its Mw value 6.75-fold in relation to the acidic biodegraded sample (Sapapporn et al., 2018). In contrast, for the acidic lignin biodegradation, a repolymerization of the lignin backbone was not indicated, which may have been caused by the methylation of phenolic hydroxyl groups in the aromatic structures of lignin (Kim et al., 2017). Regarding the lignin degradation compounds in the in the acidic and alkaline control samples after 10 days shaking, the generation of those compounds may have been caused by the auto oxidation of reactive monomers, likely related to low molar mass fractions in lignin and mediated by ABTS. For lignin biodegradation by whole bacterial cells, further studies are necessary to shed light on the synergistic reactions between laccase and putative secondary enzymes involved in lignin biodegradation (Li et al., 2014). In the case of the control samples, a better understanding of the mechanisms driving the auto oxidation reactions of the main lignin monomers is required, with vanillin as the main source of the biodegradation/auto oxidation-derived compounds.

Concerning the chemical structure of lignin, the results of FTIR analysis suggest a preponderance of G and S lignins in the samples, which is typical from softwood (Gall et al., 2014). FTIR results did not confirm the existence of H lignin. The chemical pretreatment of lignin in the acidic biodegradation method produced important modifications. The presence of the C = O bond and the OH functional group, predominantly associated with numerous carboxylic acid molecules were notable. The chemically pretreated alkali lignin with hydrogen peroxide at low pH values demonstrated its solubility in the liquid phase, without the precipitation of heavy molar mass fractions. This could be explained by the accentuation of the peak at 620 cm^–1^, which presumably was caused by a S–O bond (Nugroho Prasetyo et al., 2010). The strong peak at this wave number could indicate the lack of interference from hydrogen bonds and other non-covalent bonds at very low pH values, demonstrating that this linkage was not affected by the bacterial activity of the mutant strain *E. coli* BL21(Lacc). The alkaline biodegraded sample showed the presence of the N–H bond (from amine functional groups), confirming the character of the alkalinity of the lignin medium. The FTIR results also displayed further evidence of the existence of the C–O–C bond in the alkaline samples, a key constituent of aryl-ether linkages, viz., β–O–4 bonds. This reinforced the idea that in this case the cleavage of such bonds occurred in a lesser extent (Helmich et al., 2016). Furthermore, for the alkaline samples, the wave numbers related to aromatic skeletons/structures were more abundant, suggesting that the preponderant degradation mechanisms were not closely related to ring-opening reactions, as was the case for the acidic biodegradation sample. Table 5 summarizes the most important differences between acidic and alkaline biodegradation of lignin previously discussed.

**TABLE 5:** Summary of a general comparison between acid versus alkaline biodegradation of lignin by *E. coli* BL21(Lacc).

	Acid biodegradation	Alkaline biodegradation
Changes in OD_280_	Drop in OD_280_ values by 41.8% after 2 days	OD_280_ values rose 18.4% after 6 days
Chemical pretreatment	140°C, N_2_, 400 kPa, 141.12 mM H_2_O_2_	No chemical pretreatment
Initial/final pH values	7.0/2.53	7.0/7.90
Lignin biodegradation compounds	Crotonic acid, DL-lactic acid, 3-hydroxybutanoic acid	2-Phenylethanol, isovanillyl alcohol, dehydroabietic acid
M_w_/M_n_	4,782/2,054	32,325/7,095
Final PDI	2.32	4.55
Main chemical structure	OH and C = O for carboxylic acids C = O stretch in GS lignins, −C≡N bond Strong signal of S–O bond	OH for alcohols, C = C stretching for aromatic structures, –C≡N bond N–H bonds Stronger signals of C–O–C bonds

## Conclusion

Partial biodegradation of industrial lignins by microorganisms with ligninolytic enzymes has demonstrated strong potential for producing compounds of interest for industries seeking alternative routes for the production of chemicals. In today’s context, the synthesis of many goods still requires the use of non-renewable and toxic substrates, making the supply of more renewable compounds an urgent task. Alkali lignin is one of the industrial lignins that is produced in large amounts and can offer many opportunities due to its low valorization. The ligninolytic action of enzymes, such as bacterial laccase mediated in our case by ABTS, can further oxidize lignin monomers from low molar mass fractions, leading to the generation of many degradation compounds.

Our research strategy consisted of exploring the possibilities of the mutant bacterial strain *E. coli* BL21(Lacc) to partially degrade alkali lignin at acidic and mild alkaline conditions. In the low pH range of the spectrum, the mutant strain demonstrated its capacity to oxidize lignin monomers toward an abundant number of short-chain alkyl moieties with potential applications in the food, bio plastics, and adhesives sectors, among others. On the alkaline side of the spectrum, the metabolic activity of the bacterial mutant strain and increase in the relative abundance of compounds namely isovanillyl alcohol and dehydroabietic acid in the context of oxidative stress. Potential applications of these compounds are in the food and pharmacological industries. The possible reaction mechanism was the activation of phenyl radicals in aromatic structures, followed by substitution and repolymerization reactions, which increased its overall molar mass. This upgraded property in the alkaline biodegradation sample could render the building blocks for potential renewable substitutes in the biomaterials area.

By understanding the main reaction mechanisms involved in lignin biodegradation at different values on the pH spectrum, and by applying the appropriate chemical and biological pretreatment strategies, it is possible to unlock its potential, providing broader alternatives for renewable and bio-derived goods. Future work should tackle these issues.

## Data Availability Statement

The original contributions presented in the study are included in the article/Supplementary Material, further inquiries can be directed to the corresponding author/s.

## Author Contributions

GM conception and execution of the experiments, interpretation of the results and, writing and preparation of the manuscript and Supplementary Material. SA assistance in preliminary tests, edition of the figures and, revision and correction of the manuscript. JS and DZ funding of the research work, supervision of the experiments and, revision and corrections of the manuscript. HS assistance in the preparation of the experiments of OD, GC-MS, and SEC analyses. WZ provision of the bacterial mutant strain *E. coli* BL21(Lacc). RZ assistance in the GC-MS tests arrangements and data analysis. KH preparation of statistical analysis. All authors read and approved the final manuscript.

## Conflict of Interest

The authors declare that the research was conducted in the absence of any commercial or financial relationships that could be construed as a potential conflict of interest.

## Abbreviations

ABTS: 2,2′-azino-bis(3-ethylbenzothiazoline-6-sulfonic acid)
BSTFA-TMCS: N,O-Bis(trimethylsilyl)trifluoro-acetamide with trimethyl-chlorosilane
FTIR: Fourier-transformed infrared spectroscopy
GC-MS: gas chromatography-mass spectrometry
SEC: size exclusion chromatography
HPLC: high-performance liquid chromatography
IPTG: isopropyl ß-D-thiogalactopyranoside
LB medium: Luria and Bertani broth or also known as Lysogeny broth
mM: milli molarity
M_n_: number average molecular weight
M_w_: weight average molecular weight
NaPSS: sodium polystyrene sulfonate
OD: optical density
OH: hydroxyl
PDI: polydispersity index
pH: hydronium potential
SEM: scanning electron microscopy
TGA: thermogravimetric analysis
UV: ultraviolet radiation.
