# Corrigendum: Acidic *Versus* Alkaline Bacterial Degradation of Lignin Through Engineered Strain *E. coli* BL21(Lacc): Exploring the Differences in Chemical Structure, Morphology, and Degradation Products

**DOI:** 10.3389/fbioe.2020.00868

**Published:** 2020-08-11

**Authors:** Gabriel Murillo Morales, Sameh S. Ali, Haibing Si, Weimin Zhang, Rongxian Zhang, Keyvan Hosseini, Jianzhong Sun, Daochen Zhu

**Affiliations:** ^1^Biofuels Institute, School of Environmental Science and Safety Engineering, Jiangsu University, Zhenjiang, China; ^2^State Key Laboratory of Applied Microbiology Southern China, Guangdong Provincial Key Laboratory of Microbial Culture Collection and Application, Guangdong Open Laboratory of Applied Microbiology, Guangdong Institute of Microbiology, Guangdong Academy of Sciences, Guangzhou, China; ^3^Botany Department, Faculty of Science, Tanta University, Tanta, Egypt; ^4^School of Chemistry and Chemical Engineering, Jiangsu University, Zhenjiang, China; ^5^School of Public Affairs, University of Science and Technology of China, Hefei, China

**Keywords:** lignin, *E. coli* BL21(Lacc), biodegradation compounds, acid/alkaline incubation, depolymerization/repolymerization

In the published article, there were errors in affiliations of the authors Sameh S. Ali, Weimin Zhang and Daochen Zhu. For author Sameh S. Ali, the second affiliation should be “Botany Department, Faculty of Science, Tanta University, Tanta, Egypt” instead of “*State Key Laboratory of Applied Microbiology Southern China, Guangdong Provincial Key Laboratory of Microbial Culture Collection and Application, Guangdong Open Laboratory of Applied Microbiology, Guangdong Institute of Microbiology, Guangdong Academy of Sciences, Guangzhou, China*.” Regarding to the numbering of the affiliations, the affiliation of Botany Department, Faculty of Science, Tanta University, Tanta, Egypt, instead of having affiliation #2, it should have the affiliation #3. For author Weimin Zhang, the affiliation should be “*State Key Laboratory of Applied Microbiology Southern China, Guangdong Provincial Key Laboratory of Microbial Culture Collection and Application, Guangdong Open Laboratory of Applied Microbiology, Guangdong Institute of Microbiology, Guangdong Academy of Sciences, Guangzhou, China*” instead of “*Botany Department, Faculty of Science, Tanta University, Tanta, Egypt*.” For author Daochen Zhu, the second affiliation should be “*State Key Laboratory of Applied Microbiology Southern China, Guangdong Provincial Key Laboratory of Microbial Culture Collection and Application, Guangdong Open Laboratory of Applied Microbiology, Guangdong Institute of Microbiology, Guangdong Academy of Sciences, Guangzhou, China*” instead of *Botany Department, Faculty of Science, Tanta University, Tanta, Egypt*. Regarding to the numbering of the affiliations, the affiliation of the *State Key Laboratory of Applied Microbiology Southern China, Guangdong Provincial Key Laboratory of Microbial Culture Collection and Application, Guangdong Open Laboratory of Applied Microbiology, Guangdong Institute of Microbiology, Guangdong Academy of Sciences, Guangzhou, China*, instead of having affiliation #3, it should have the affiliation #2.

Also, in the article's citation, an author name was incorrectly presented as Morales GM. The correct spelling is Murillo Morales G.

Additionally, Figures 1–5 were presented in a wrong order. Figure 1 should be **Figure 5**, Figure 2 should be Figure 1, Figure 3 should be Figure 2, Figure 4 should be Figure 3, and Figure 5 should be Figure 4.

**Figure 1:**
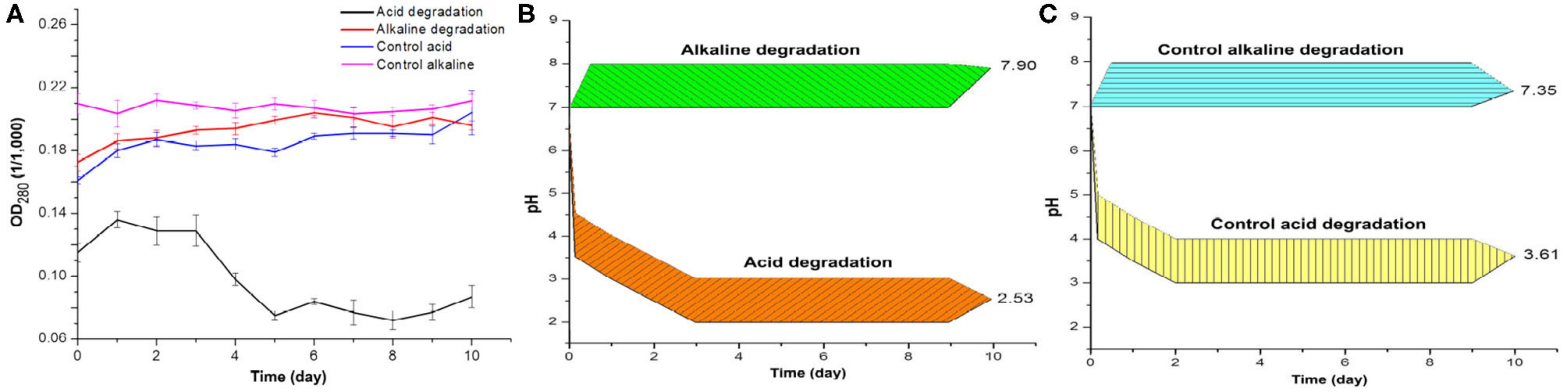
**(A)** Optical density of acid and alkaline-biodegraded lignin and controls at 280 nm. **(B)** pH measures of acid and alkaline-biodegraded lignin and **(C)** pH measures of controls. The range of the pH measures is ±0.50.

**Figure 2:**
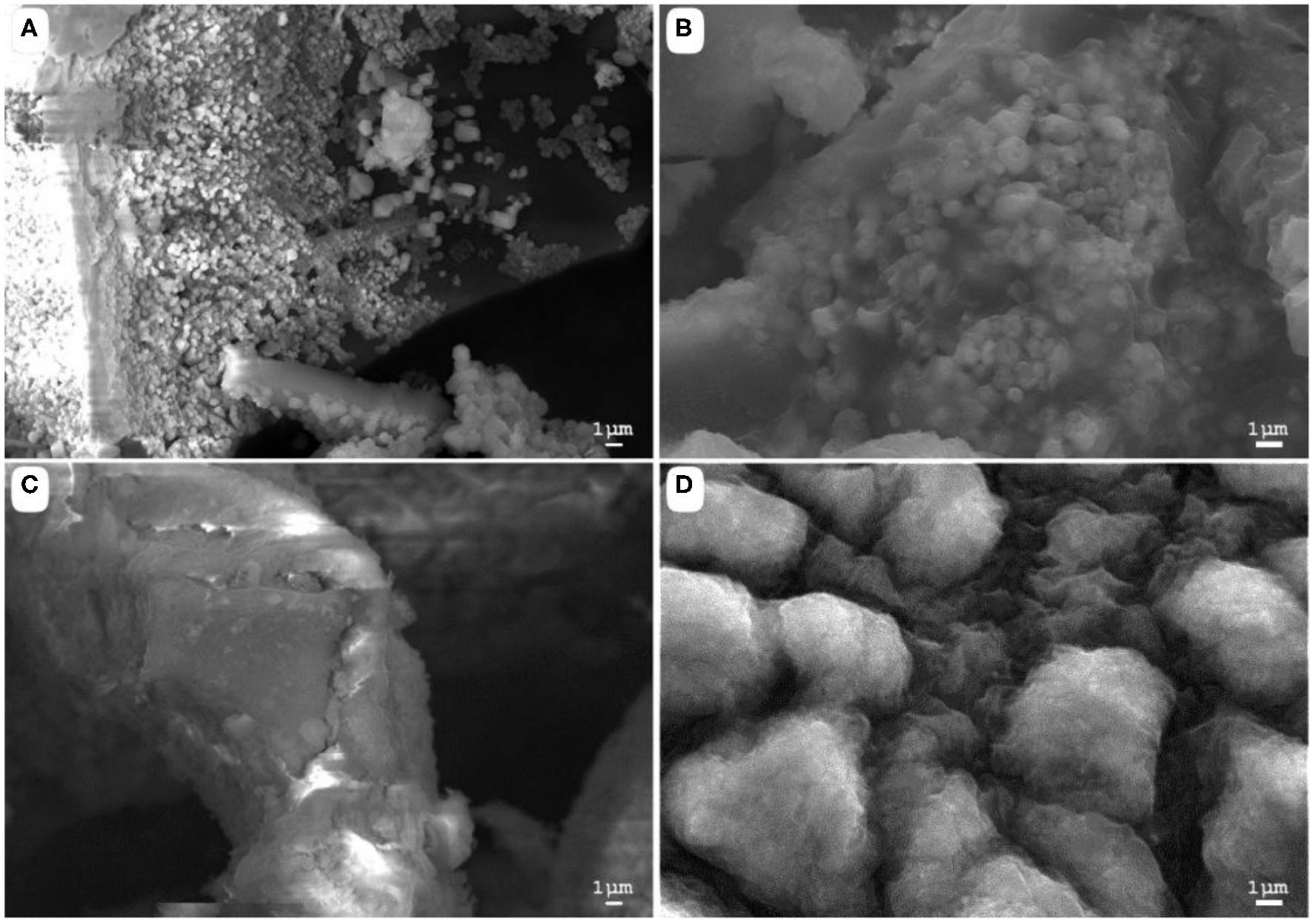
Scanning electron microscopy images of **(A)** acid-biodegraded lignin, **(B)** alkaline-biodegraded lignin, **(C)** control of acid biodegradation, and **(D)** control of alkaline biodegradation. Scale of reference of 1 μm.

**Figure 3:**
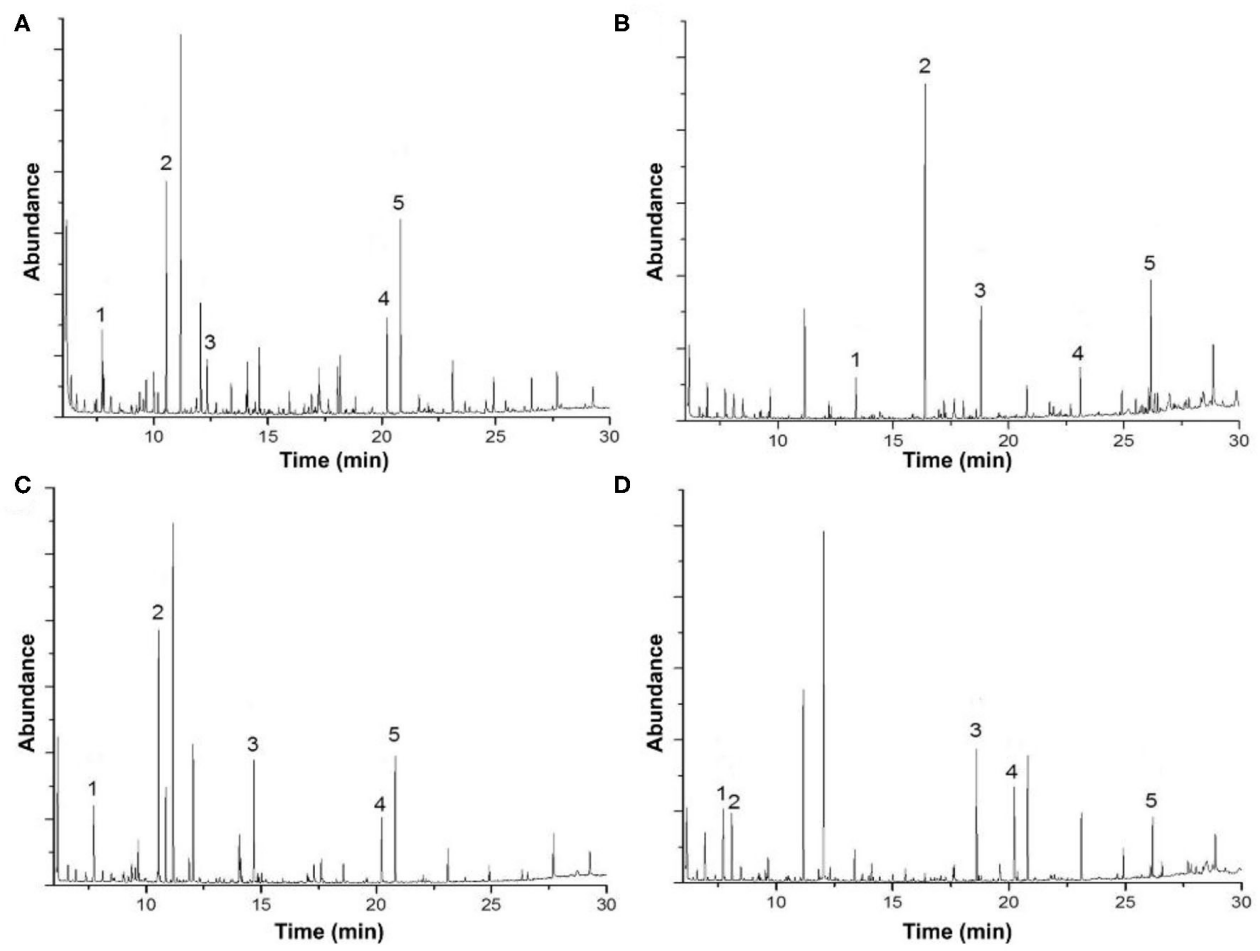
Gas chromatography-mass spectrometry analysis of **(A)** acid biodegradation of lignin, **(B)** alkaline biodegradation of lignin, **(C)** control for acid biodegradation, and **(D)** control for alkaline biodegradation.

**Figure 4:**
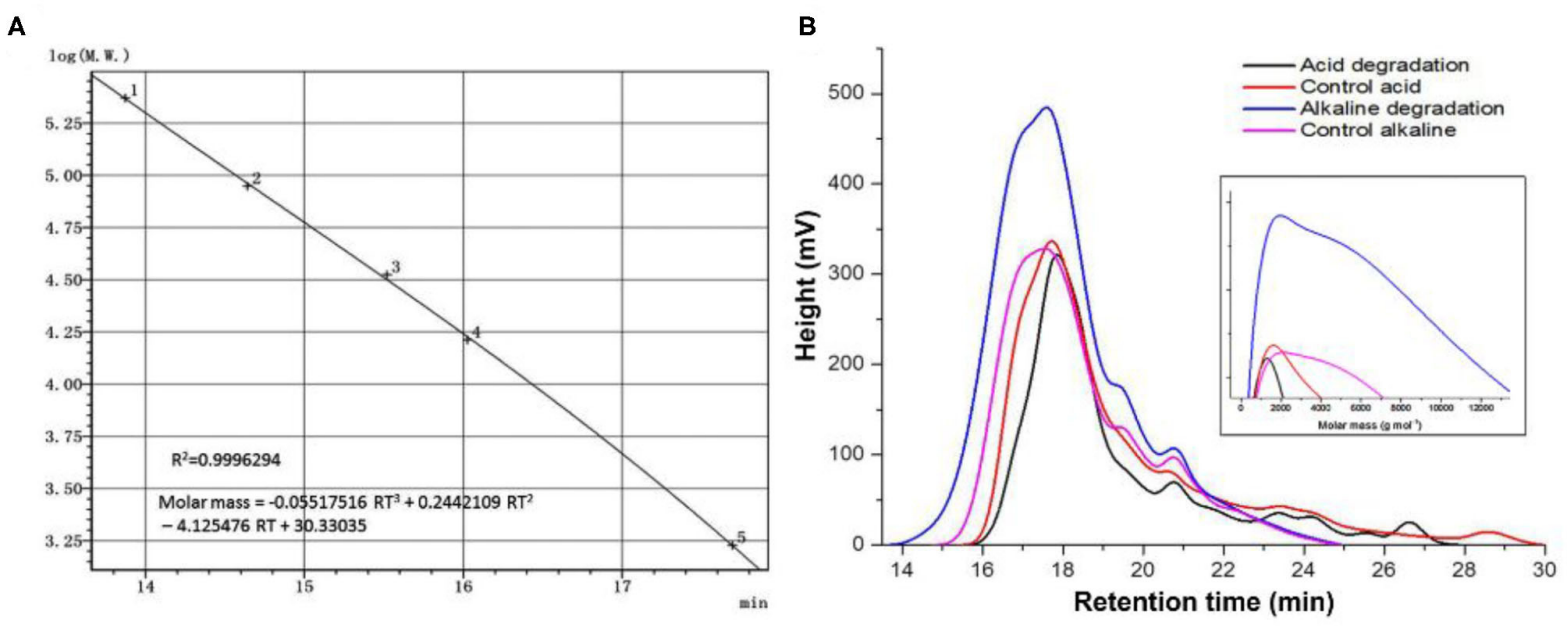
Size exclusion chromatography analysis. **(A)** Universal calibration, **(B)** curves of acid, alkaline biodegradation of lignin, and controls. From the equation of the universal calibration, “RT” means “retention time.”

**Figure 5:**
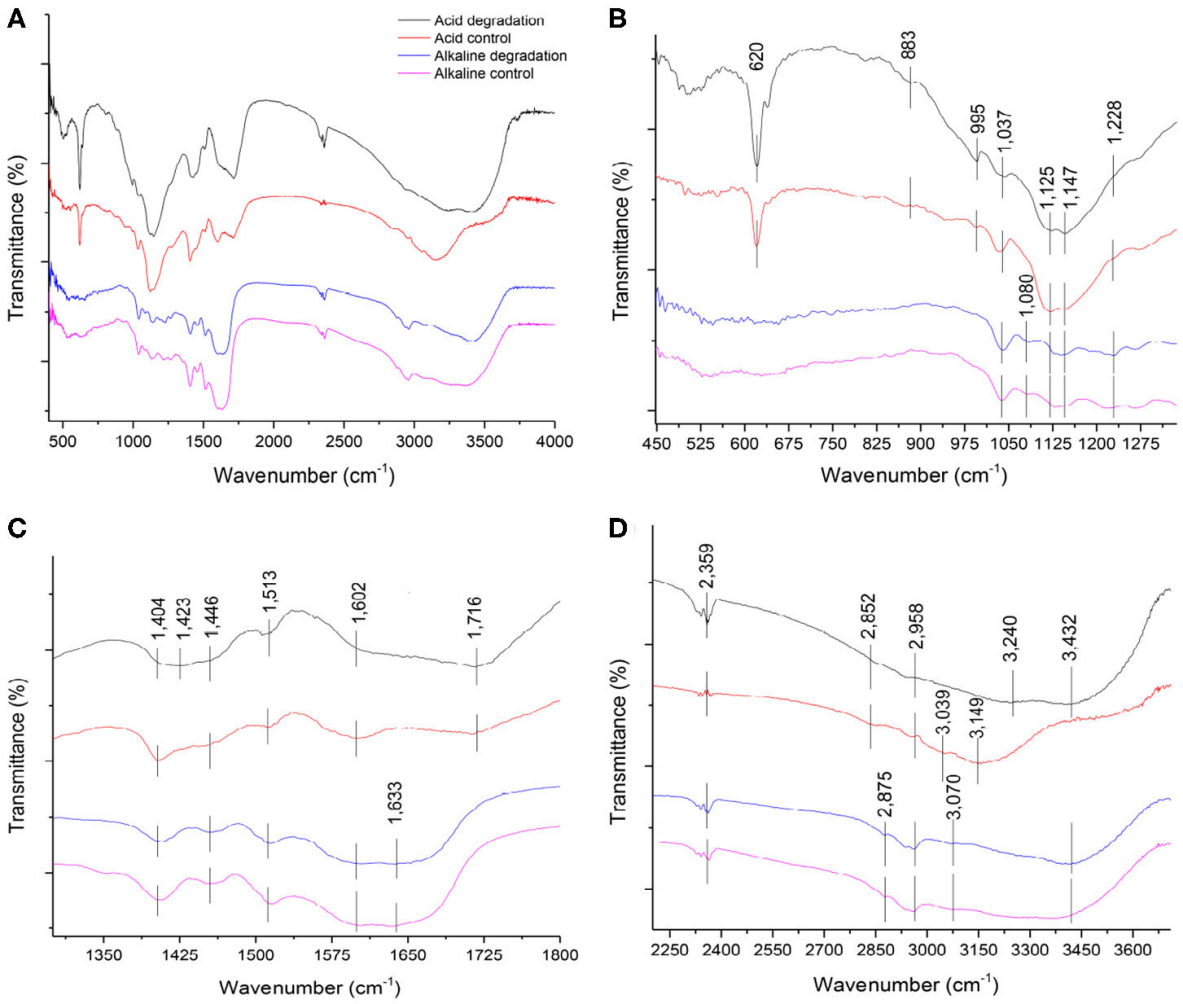
**(A)** Overall FTIR spectra of acid and alkaline biodegradation of lignin and controls; **(B)** spectra from wave numbers 450–1,300 cm^−1^; **(C)** 1,300–1,800 cm^−1^; **(D)** 2,250–3,700 cm^−1^.

There was also an error in the abstract text. The name of the mutant bacterial strain was incorrectly written as *E.coli* BL21 (Laccase). Instead, it should be written as *E.coli* BL21 (Lacc). A correction has been made to abstract, last sentence:

“Lignin biodegradation products from *E.coli* BL21 (Lacc), under different initial pH conditions, demonstrated a promising potential to enlarge the spectrum of renewable products for biorefinery activities.”

The authors apologize for these errors and state that they do not change the scientific conclusions of the article in any way. The original article has been updated.

