# Supplementary material for: Acidic Versus Alkaline Bacterial Degradation of Lignin Through Engineered Strain E. coli BL21(Lacc): Exploring the Differences in Chemical Structure, Morphology, and Degradation Products
Source: Front Bioeng Biotechnol. 2020 Jun 30;8:671. doi: 10.3389/fbioe.2020.00671 (PMC7344149; doi:10.3389/fbioe.2020.00671)
Supplement: Supplementary file 1 [file Data_Sheet_1.docx]

Fig SM #1. SEM image of alkali lignin without chemical/biological treatment.
