# Supplementary material for: Acidic Versus Alkaline Bacterial Degradation of Lignin Through Engineered Strain E. coli BL21(Lacc): Exploring the Differences in Chemical Structure, Morphology, and Degradation Products
Source: Front Bioeng Biotechnol. 2020 Jun 30;8:671. doi: 10.3389/fbioe.2020.00671 (PMC7344149; doi:10.3389/fbioe.2020.00671)
Supplement: Supplementary file 2 [file Data_Sheet_2.docx]

Supplementary file #2: GC/MS analysis for acid and alkaline biodegradation of lignin and their controls.

Table SM 1A GC-MS analysis for acidic biodegradation of lignin

| Retention time | Name of compound | Probability | Relative  abundance |
| --- | --- | --- | --- |
| (min) |  |  |  |
| 6.157 | Trisiloxane, octamethyl- | 90% | 4.68% |
| 6.358 | Acetoin | 64% | 2.11% |
| 7.724 | Crotonic acid | 78% | 5.21% |
| 7.784 | 3-Methylbutanoic acid | Confirmed | 2.23% |
| 9.662 | 1,4-Dioxan-2-ol | 93% | 2.14% |
| 9.994 | Butane-2,3-diol | Confirmed | 2.45% |
| 10.536 | DL-lactic acid | 90% | 11.27% |
| 11.169 | Ethyl(dimethyl)ethoxysilane | 39% | 20.22% |
| 12.042 | 2-Methylbutan-1-ol | 35% | 6.21% |
| 12.334 | 3-Hydroxybutanoic acid | Confirmed | 3.32% |
| 13.388 | 2-Phenylethanol | Confirmed | 1.53% |
| 14.101 | Glycerol | 90% | 2.51% |
| 14.624 | Pentan-2-ol | 47% | 3.15% |
| 17.235 | Adipic acid | Confirmed | 2.29% |
| 18.058 | 2-Hydroxy-2-isopropylsuccinic acid | 74% | 3.07% |
| 18.179 | 3-Phenyllactic acid | 87% | 3.31% |
| 20.228 | Vanillic acid | 99% | 4.83% |
| 20.82 | 3,4-Dihydroxybenzoic acid | 99% | 9.66% |
| 23.11 | Hexadecanoic acid | 99% | 2.83% |
| 24.908 | Octadecanoic acid | 99% | 2.16% |
| 26.585 | Butanoic acid, 2-(acetylamino)-4-cyano-, |  |  |
|  | 2,6-bis(1,1-dimethylethyl)-4-methoxyphenyl ester | 50% | 2.07% |
| 27.7 | 1H-Pyrrole, 2,3,4,5-tetraphenyl- | 59% | 2.63% |

Table SM 1B GC-MS analysis for alkaline biodegradation of lignin

| Retention time | Name of the compound | Probability | Relative abundance |
| --- | --- | --- | --- |
| (min) |  |  |  |
| 6.165 | Trisiloxane, octamethyl- | 91% | 7.05% |
| 6.942 | 2,2,2-Trifluoroacetamide | 53% | 3.96% |
| 11.164 | Ethyl(dimethyl)ethoxysilane | 39% | 10.65% |
| 13.386 | 2-Phenylethanol | 90% | 3.75% |
| 16.386 | 1H-Indole | 94% | 32.00% |
| 18.808 | Isovanillyl alcohol | 99% | 10.26% |
| 20.796 | Pyrrolo[1,2-a]pyrazine-1,4-dione, |  |  |
|  | hexahydro-3-(2-methylpropyl)- | 47% | 4.50% |
| 23.107 | Hexadecanoic acid | 99% | 4.33% |
| 26.173 | Dehydroabietic acid | 95% | 13.43% |
| 28.873 | Methyl 2-hydroxy-2-phenylacetate | 64% | 10.04% |
|  |  |  |  |

Table SM 1C GC-MS analysis of control for acidic biodegradation of lignin

| Retention time | Name of the compound | Probability | Relative  abundance |
| --- | --- | --- | --- |
| (min) |  |  |  |
|  |  |  |  |
| 6.167 | Trisiloxane, octamethyl- | 91% | 11.82% |
| 7.734 | Crotonic acid | 83% | 6.36% |
| 9.662 | 1,4-Dioxan-2-ol | 87% | 2.76% |
| 10.55 | Propylene glycol | 72% | 16.48% |
| 10.86 | 2-Hydroxyacetic acid | 91% | 5.94% |
| 11.17 | Ethyl(dimethyl)ethoxysilane | 39 | 22.74 |
| 12.05 | 4-Methylmannitol | 45% | 9.22% |
| 14.05 | Silanol, trimethyl-, phosphate (3:1) | 99 | 2.85 |
| 14.68 | Succinic acid | 98% | 7.10% |
| 20.23 | Vanillic acid | 96% | 4.34% |
| 20.82 | Protocatechuic acid | 99% | 7.88% |
| 23.11 | Hexadecanoic acid | 99 | 2.50% |
|  |  |  |  |

Table SM 1D GC-MS analysis of control for alkaline biodegradation of lignin

| Retention time | Name of the compound | Probability | Relative abundance |
| --- | --- | --- | --- |
| (min) |  |  |  |
|  |  |  |  |
| 6.165 | Trisiloxane, octamethyl- | 91% | 5.64% |
| 6.942 | 2,2,2-Trifluoroacetamide | 49% | 4.24% |
| 7.731 | Crotonic acid | 83% | 5.46% |
| 8.098 | Ethanamine | 93% | 5.91% |
| 9.664 | 1,4-Dioxan-2-ol | 90% | 1.78% |
| 11.164 | 1,3-Oxathiane, 2-(1-methylethyl)- | 39% | 13.49% |
| 12.042 | 2,2'-Bi-1,4-dioxane | 33% | 21.02% |
| 13.375 | Guaiacol | 96% | 2.18% |
| 18.608 | Apocynin | 99% | 8.53% |
| 20.23 | Vanillic acid | 96% | 6.11% |
| 20.807 | Hydroconiferyl alcohol | 91% | 9.54% |
| 23.107 | Hexadecanoic acid | 99% | 4.76% |
| 24.907 | Octadecanoic acid | 99% | 2.11% |
| 26.173 | Dehydroabietic acid | 95% | 4.47% |
| 28.873 | 2',4'-Dimethoxy-3'-methylpropiophenone | 47% | 4.73% |
|  |  |  |  |
