# Supplementary material for: Acidic Versus Alkaline Bacterial Degradation of Lignin Through Engineered Strain E. coli BL21(Lacc): Exploring the Differences in Chemical Structure, Morphology, and Degradation Products
Source: Front Bioeng Biotechnol. 2020 Jun 30;8:671. doi: 10.3389/fbioe.2020.00671 (PMC7344149; doi:10.3389/fbioe.2020.00671)
Supplement: Supplementary file 3 [file Data_Sheet_3.docx]

Table SM 2. Changes in the relative abundance of 1H-indole, dehydroabietic acid and isovaillyl alcohol

| Compounds/lignin metabolites | | | | |
| --- | --- | --- | --- | --- |
|  | Days | | | |
|  | 0 | 3 | 7 | 10 |
| 1H-Indole | 19.71% | 21.60% | 56.52% | 32.00% |
| Dehydroabietic acid | 2.70% | 5.23% | 13.14% | 13.44% |
| Isovanillyl alcohol | 3.96% | 4.64% | 12.10% | 10.26% |
|  |  |  |  |  |
